# The Constitution of the Hospital Sunday Fund

**Published:** 1904-12-31

**Authors:** 


### THE CONSTITUTION OF THE HOSPITAL SUNDAY FUND.

The *Medical Press*, in its issue of the 21st inst., referring to the Metropolitan Hospital Sunday Fund, declares "Its constitution is directly opposed to the enlightened and democratic tendencies of the age." We wonder if the writer in our contemporary has ever read the laws of the constitution of the Sunday Fund? The money entrusted to its council is given by the congregations of all denominations. These congregations represent every class and every creed of Londoners. They collect the money, and the constitution of the Fund provides that every congregation which has forwarded a contribution during two successive years shall be entitled to a voice in the management. Each year the minister and two laymen, representing every such congregation, are summoned to the Mansion House to receive the annual report, and to elect the council for the succeeding year. The council consists of not more than 50 clerical and 50 lay members, in addition to the President, the Vice-President, the members of the Committee of Distribution, and the honorary secretaries who are ex officio members of the council. Such is the constitution of the Hospital Sunday Fund, and it will be interesting to learn from the *Medical Press* in what respects and in what way it is "opposed to the enlightened and democratic tendencies of the age?" If the people who subscribe and collect the funds are not the right people to control the government of the Fund and its distribution, "in accordance with the enlightened and democratic tendencies of the age," where are they to be found?

